# Experiences of coercion amongst involuntary mental health care users in KwaZulu-Natal, South Africa

**DOI:** 10.3389/fpsyt.2023.1113821

**Published:** 2023-03-07

**Authors:** Zinhle Shozi, Shamima Saloojee, Sibongile Mashaphu

**Affiliations:** Department of Psychiatry, School of Clinical Medicine, College of Health Sciences, University of KwaZulu-Natal, Durban, South Africa

**Keywords:** involuntary, admission experience, coercion, psychiatry, South Africa

## Abstract

**Background:**

Involuntary admission is a common practice globally. Previous international studies reported that patients experienced high levels of coercion, threats and a range of negative emotions. Little is known about the patients’ experience in South Africa. The aim of this study was to describe the patient’s experiences of involuntary admission at two psychiatric hospitals in KwaZulu-Natal.

**Methods:**

A cross-sectional descriptive quantitative study of patients admitted involuntarily was conducted. Demographic information was extracted from clinical records and interviews were conducted with consenting participants at discharge. The MacArthur Perceived Coercion Scale, the MacArthur Negative Pressures Scale, and the MacArthur Procedural Justice Scale, of the MacArthur Admission Experience Survey (short form) were utilized to describe participants’ experiences.

**Results:**

This study comprised 131 participants. The response rate was 95.6%. Most participants (*n* = 96; 73%) experienced high levels of coercion and threats (*n* = 110; 84%) on admission. About half (*n* = 61; 46.6%) reported that they felt unheard. Participants reported feeling sad (*n* = 68; 52%), angry (*n* = 54; 41.2%), and confused (*n* = 56; 42.7%). There was a significant association between good insight and a feeling of relief (*p* = 0.001), and between poor insight and feelings of anger (*p* = 0.041).

**Conclusion:**

The findings of this study confirm that most patients who were admitted involuntarily experienced high levels of coercion, threats, and exclusion from the decision-making process. Patient involvement and control of the decision-making process must be facilitated to improve clinical and overall health outcomes. The need for involuntary admission must justify the means.

## Introduction

The use of coercion in psychiatry is controversial because persuading someone to be admitted to a hospital using force or threats infringes on an individual’s right to autonomy and freedom of movement (1, 2). Coercion can be formal or informal. Formal coercion refers to forms of coercion allowed by law, including involuntary admission, physical restraint, and forced pharmacological therapy (3, 4). Informal coercion is defined as the use of techniques such as persuasion, inducement, and interpersonal leverage to encourage admission (5).

It is debatable whether the benefit to a patient outweighs the risks associated with an admission that is coerced (1). Furthermore, there is good evidence from several international studies to show that an involuntary admission in psychiatry evokes a wide range of negative emotions (6–12), including anger, rage, despair (6) and confusion (13). An involuntary admission has been described as a frightening (9) and a “life-changing experience” (11), with patients reporting feeling trapped, disengaged, disempowered and unsupported at various stages of their admission (10).

The MacArthur Admission Experience Survey (MAES) (short form) has been used by previous authors to document patients’ experiences of their involuntary admission (13–15). The experiences are grouped into three clusters: (i) feelings of coercion or perceived coercion, (ii) negative pressures, and (iii) voice or procedural justice. Perceived coercion refers to an individual’s experience of being pressured to enter treatment (16), and can be psychological with an external or internal source (4). Negative pressure refers to the psychological or physical pressure that was exerted on the individual at the time of admission (17). Procedural justice refers to fairness, respect, and transparency regarding the decision-making process (18).

Previous research shows that factors associated with high levels of perceived coercion included involuntary admission status, female gender, insight, seclusion, and restraint (4, 19, 20). Negative pressure scores in previous studies were significantly associated with involuntary admission status, positive symptoms of schizophrenia (19), male gender, a longer duration of illness, previous forensic history, and individuals who came from extended family structures (21), whilst perceived procedural injustice was significantly associated with fewer negative psychotic symptoms, female gender, involuntary status, and cognitive impairment (19).

Possible adverse consequences following an involuntary admission include stigma (22), a negative therapeutic relationship (9), poorer quality of life, longer length of hospitalization, increased number of aggressive incidents and dissatisfaction with treatment (2). Despite the negative experience associated with involuntary admission, mental health laws globally permit coercive admission for the treatment of patients with mental illnesses to restore health and prevent harm to self or others.

As is the case with mental health legislation worldwide, the procedure for the admission of involuntary patients in South Africa (SA) has been carefully drafted (23, 24). According to the Mental Health Care Act (MHCA) no. 17 of 2002, patients can be admitted to a health establishment without their consent on the grounds of mental illness under two sections: section 26 for the care of assisted mental health care users and section 33 for the care of involuntary mental health care users. Involuntary care is defined as the provision of health interventions to people incapable of making informed decisions due to their mental health status, and who refuse health intervention, but require such services for their own protection, or the protection of others. An application for involuntary admission is the first step in the process. The patient must then be examined by two health care workers. One of these two must be qualified to do a physical examination. If it is found that the patient is indeed ill with a mental health condition, then the head of the health establishment will issue an order for the patient to be admitted involuntarily to that establishment (24).

However, in the face of a high patient burden, a lack of trained mental health care workers and inadequate resource allocation for mental health, the implementation of mental health law in SA has been challenging (25, 26). For example, although a patient has a right to appeal against his/her involuntary admission, this procedure is delayed by poorly functioning Mental Health Review Boards (23). Previous research has found that Mental Health Review Boards do not visit hospitals frequently, do not communicate rulings timeously (27) and have not been held to account when the rights of mental health care users were disregarded (28).

Involuntary admission is a common practice in SA (27, 29), and a recent study from a psychiatric hospital in SA found that involuntary patients were more likely to have dignity-related complaints compared to voluntary patients (30). It is also important to prevent a negative involuntary admission experience because there is a suggestion that an involuntary admission does not necessarily have to bring about a feeling of coercion (31).

However, there is a dearth of information regarding the patient’s experience of involuntary admission in SA. In this study, we, therefore, aim to describe the patients’ experience of involuntary admission and to discuss the possible associations of perceived coercion with sociodemographic and clinical factors. We also measured patient insight and examined possible associations with experiences of coercion. This study was restricted to involuntary patients because there is a shortage of psychiatric beds in the region leading to hopelessly overcrowded wards. Hence most admissions to the psychiatric hospitals are either involuntary or assisted (27). Gaining an understanding of the involuntary patient experience in SA will assist in the development of policies to enhance the care and treatment of patients who are coerced into admission.

## Materials and methods

### Study design

This was a descriptive quantitative, cross-sectional study. Information was gathered from a chart review combined with a structured patient interview at discharge.

### Study location

This study was conducted at King Dinuzulu Hospital Complex and Town Hill Hospital in KwaZulu-Natal, South Africa. King Dinuzulu Hospital Complex is in Sydenham, Durban. This public hospital offers mental health services at both a district and specialized level to patients who live in the surrounding area. Patients are referred to the specialized psychiatric service from all the general hospitals in area one of KwaZulu Natal. Townhill is a public hospital in Pietermaritzburg that provides specialized psychiatric services for areas two and three of KwaZulu Natal.

### Study population and sampling strategy

Involuntary patients above the age of 18 years who were admitted at the study sites under section 33 of the MHCA 17 of 2002 were informed about the study by the treating medical practitioner upon discharge from the ward. Patients who were willing to participate in the study were then referred to the principal investigator (PI). Patients were interviewed by the PI independently. Participants who could provide informed consent were recruited for the study by the PI. The treating medical practitioner and PI emphasized that participation in the study had no bearing on the current and future care and treatment of the patient. Patients diagnosed with a neurocognitive disorder or intellectual disability were excluded. The study participants were selected from the total number of involuntary admissions admitted over a 6-month period using nonprobability purposive sampling.

### Data collection methods and tools

Patients were interviewed using two scales. The MAES was used to assess the admission experience, and the Birchwood Insight Scale was used to measure insight. The MAES has been validated for use in America, Europe, China, and French-speaking countries (14, 17, 32, 33). Although the MAES (short form) has not been validated in SA, it has been used to investigate the admission experience of involuntary patients in other low and middle-income countries such as Pakistan (13) and India (34).

Interviews were conducted in English. The principal investigator is fluent in English and Isizulu and she was therefore able to provide clarity for those patients whose first language was not English. Demographic and clinical information was extracted from the clinical records and MHCA forms at discharge.

The MAES (short form) is a 16-item questionnaire that allows for the computation of four subscales and a total score. The MAES measures perceived coercion, negative pressures, procedural injustice, and affective reactions. In this study only the first three of the four subscales were scored. The MacArthur Perceived Coercion Scale (MPCS) is a measure of freedom of choice and initiative around the admission (16). The MacArthur Negative Pressures Scale (MNPS) is a measure of the use of force or threats during the admission treatment, whilst the MacArthur Procedural Justice Scale (MPJS) is a measure of the participant’s perception about the opportunity to voice their opinion regarding hospitalization (33).

The MPCS is scored on a scale from 0 to 5, the MNPS, is scored from 0 to 6, and the MPJS, is scored from 0 to 3. In this study a score of ≥3 on the MPCS was classified as high perceived coercion and a score of ≥4 on the MNPS was considered as high negative pressures. A score of 0–1 on the MPJS was considered as high procedural injustice as lower scores indicate higher injustice. This classification is in keeping with international studies utilizing the same scale.

The Birchwood insight scale (BIS) is an 8-item scale measuring insight with a total possible score of 0–16 (35). The BIS has been used in South African studies to assess insight in Schizophrenia (36, 37). A score of 9 or more was classified as good insight (35).

### Data analysis techniques

The data was entered into a REDCAP database (38). STATA SE version 17 was used to analyze data. Descriptive statistics are presented using frequencies, percentages, and measures of central tendencies. We dichotomized scores of the MAES and the BIS into high and low scores as explained in the methods section. Pearson’s Chi-squared test was used to test for association between dichotomous sociodemographic variables and dichotomized MAES and BIS scores. The Fischer’s exact test was used where groups in the categorical data had frequencies of less than five. The Wilcoxon rank-sum was used to test for association between non-parametric continuous data and categorical data.

### Ethical considerations

Permission was granted by the University of KwaZulu-Natal Biomedical Research Ethics Committee, approval number BREC/00000208/2019. The principal investigator also acquired permission from the Head of The Health Establishments of the hospitals and the KwaZulu Natal department of health. All participants provided written, informed consent. Data collected was stored in a password-protected REDCAP database. Only the principal investigator had access to identifying patient information.

## Results

Of the 137 patients who were approached to participate in the study, 131 consented. The response rate was 95.6%. The median age of the sample was 28 years (IQR = 23–37). Most participants were male (*n* = 87; 66.4%), black African (*n* = 102, 77.6%), single (*n* = 112; 85.5%), unemployed (*n* = 95; 72.52%), and had a secondary level of education (*n* = 46; 35.11%). More than half of the patients (*n* = 71; 54.19%) passed matric. Only three (2.29%) participants were not literate (Table 1).

**Table 1:** Demographic information.

Variable		*N* (131)	%
**Age, median (IQR)**		28.45 (23.99, 37.25)	
**Gender**	Female	44	33.59%
	Male	87	66.41%
**Ethnicity**	African	102	77.86%
	Mixed-race	11	8.40%
	White	5	3.82%
	Indian	12	9.16%
	Asian	1	0.76%
**Home language**	IsiZulu	94	71.76%
	English	27	20.61%
	Afrikaans	2	1.53%
	Other	8	6.11%
**Marital status**	Single	112	85.5%
	Married	10	7.69%
	Divorced	7	5.38%
	Cohabiting	1	0.77%
	Other	1	0.77%
**Number of children, median (IQR)**		1.00 (0.00, 2.00)	
**Type of housing**	Informal	17	12.98%
	Formal	114	87.02%
**Highest level of education**	No education	3	2.29%
	Primary school education	11	8.40%
	High school education	46	35.11%
	Matric	37	28.24%
	Tertiary incomplete	17	12.98%
	Tertiary completed	17	12.98%
**Employment status**	Unemployed	95	72.52%
	Student	7	5.34%
	Employed	21	16.03%
	Self-employed	8	6.11%
**Disability grant**	No	107	81.68%
	Yes	24	18.32%
**Previously arrested**	No	103	78.63%
	Yes	28	21.37%
**Use of substances**	No	32	24.43%
	Yes	99	75.57%
**Type of substance used**	Tobacco	20	20.41%
	Alcohol	14	14.29%
	Cannabis	32	32.65%
	Other	32	32.65%
**Diagnosis**	Psychotic disorders	104	79.39%
	Mood disorders	14	10.69%
	Other	13	9.92%

IQR, inter quartile range.

In this sample, most of the participants had a diagnosis of a psychotic disorder, which includes schizophrenia (*n* = 52; 39.7%), schizoaffective disorder (*n* = 19; 14.5%), substance-induced psychotic disorder (*n* = 14; 10.7%), psychotic disorder due to another medical condition (*n* = 13; 9.9%), unspecified schizophrenia spectrum, and other psychotic disorders (*n* = 6; 4.6%). In the “other” category, six participants (4.5%) were admitted mainly for a non-psychotic unipolar depressive disorder, three (2.29%) for adjustment disorder, two (1.52%) for borderline personality disorder crises, and two (1.52%) for substance intoxication or withdrawal (Table 1). Most participants (73.3%) had to be sedated during admission, with just over a third (35%) of the participants having been brought in by the police. The median length of admission was 17 days (IQR 9–69).

Over two-thirds of the participants stated that it was not their idea to come into the hospital (*n* = 102; 77.9%) and that they did not have a lot of control over whether they went to the hospital or not (*n* = 92; 70.2%). One in two participants admitted to feeling sad (*n* = 68; 51.9%), 42.7% (*n* = 56) reported that they were confused, and 41.2% (*n* = 54) were angry about the involuntary admission. Confused, in this instance, refers a state of not being clear about what is happening regarding the involuntary admission process (39). About one in three participants (*n* = 48; 36.6%) reported being fearful. However, at discharge more than half of the participants stated that they felt relieved about the current admission (*n* = 76; 58%), and one-third (*n* = 48; 36.6%) were pleased regarding the admission (Table 2).

**Table 2:** Experience of the current admission using the MAES.

Question	Participant answer	*N* = 131	%
Angry	False	75	57.3%
	True	54	41.2%
	Do not know	2	1.5%
Sad	False	62	47.3%
	True	68	51.9%
	Do not know	1	0.8%
Pleased	False	81	61.8%
	True	48	36.6%
	Do not know	2	1.5%
Relieved	False	51	38.9%
	True	76	58.0%
	Do not know	4	3.1%
Confused	False	73	55.7%
	True	56	42.7%
	Do not know	2	1.5%
Frightened	False	82	62.6%
	True	48	36.6%
	Do not know	1	0.8%

The proportion of participants with high scores on the MPCS (≥3), MNPS (≥4), and MPJS (0–1) was 73.3% (*n* = 96), 84% (*n* = 110), and 46.6% (*n* = 61) respectively. Table 3 shows the associations between various sociodemographic variables and the MAES sub-scores. There was no statistically significant association between age, gender, race, marital status, level of education, diagnosis, and employment and the three subscales of the MAES.

**Table 3:** Associations between MAES and sociodemographic factors.

Coercion				Negative pressure		Procedural injustice				
Level *n* (%)	Low	High	*p*-Value	Low	High	*p*-Value	High	Low	*p*-Value	Test
	35 (26.7%)	96 (73.2%)		21 (16.0%)	110 (83.9%)		61 (46.6%)	70 (53.4%)		
**Age**	26.0 (23.0, 28.0)	27.0 (24.0, 33.0)	0.51	24.0 (20.0, 30.0)	27.0 (24.0, 33.0)	0.30	24.0 (23.0, 30.0)	27.0 (24.0, 33.0)	0.53	Wilcoxon rank-sum
**Gender:** Female	11 (31%)	33 (34%)	0.75	7 (33.3%)	37 (33.6%)	0.98	21 (34%)	23 (33%)	0.85	Pearson’s chi-squared
Male	24 (69%)	63 (66%)		14 (66.7%)	73 (66.4%)		40 (66%)	47 (67%)		
**Ethnicity:** African	26 (74%)	76 (79%)	0.35	16 (76.2%)	86 (78.2%)	0.93	47 (77%)	55 (79%)	0.58	Fisher’s exact
Mixed-race	5 (14%)	6 (6%)		2 (9.5%)	9 (8.2%)		6 (10%)	5 (7%)		
White	0 (0%)	5 (5%)		1 (4.8%)	4 (3.6%)		1 (2%)	4 (6%)		
Indian	4 (11%)	8 (8%)		2 (9.5%)	10 (9.1%)		7 (11%)	5 (7%)		
Asian	0 (0%)	1 (1%)		0 (0.0%)	1 (0.9%)		0 (0%)	1 (1%)		
**Marital status**: Single	30 (86%)	82 (85%)	1.00	18 (85.7%)	94 (85.5%)	0.50	50 (82%)	62 (89%)	0.13	Fisher’s exact
Married	3 (9%)	7 (7%)		3 (14.3%)	7 (6.4%)		5 (8%)	5 (7%)		
Divorced	2 (6%)	5 (5%)		0 (0.0%)	7 (6.4%)		6 (10%)	1 (1%)		
Cohabiting	0 (0%)	1 (1%)		0 (0.0%)	1 (0.9%)		0 (0%)	1 (1%)		
Other	0 (0%)	1 (1%)		0 (0.0%)	1 (0.9%)		0 (0%)	1 (1%)		
**Employment status:** Employed	27 (77%)	68 (71%)	0.22	16 (76.2%)	79 (71.8%)	1.00	50 (82%)	45 (64%)	0.11	Fisher’s exact
Student	1 (3%)	6 (6%)		1 (4.8%)	6 (5.5%)		1 (2%)	6 (9%)		
Employed	3 (9%)	18 (19%)		3 (14.3%)	18 (16.4%)		7 (11%)	14 (20%)		
Self-employed	4 (11%)	4 (4%)		1 (4.8%)	7 (6.4%)		3 (5%)	5 (7%)		
**Diagnosis:** Psychotic disorder	28 (80%)	76 (79%)	0.076	16 (76.2%)	88 (80.0%)	0.83	48 (79%)	56 (80%)	0.44	Fisher’s exact
Mood disorder	1 (3%)	13 (14%)		3 (14.3%)	11 (10.0%)		5 (8%)	9 (13%)		
Other	6 (17%)	7 (7%)		2 (9.5%)	11 (10.0%)		8 (13%)	5 (7%)		
**Educational level:** No education	0 (0%)	3 (3%)	0.99	0 (0.0%)	3 (2.7%)	0.38	0 (0%)	3 (4%)	0.17	Fisher’s exact
Primary school education	3 (9%)	8 (8%)		0 (0.0%)	11 (10.0%)		8 (13%)	3 (4%)		
High school education	13 (37%)	33 (34%)		10 (47.6%)	36 (32.7%)		23 (38%)	23 (33%)		
Matric	10 (29%)	27 (28%)		8 (38.1%)	29 (26.4%)		18 (30%)	19 (27%)		
Tertiary education incomplete	5 (14%)	12 (13%)		1 (4.8%)	16 (14.5%)		5 (8%)	12 (17%)		
Tertiary education complete	4 (11%)	13 (14%)		2 (9.5%)	15 (13.6%)		7 (11%)	10 (14%)		

Regarding insight at discharge, most patients (*n* = 113; 86.3%) reported that they were mentally well, and that their recent stay in the hospital was necessary (*n* = 81; 61.8%). Around two-thirds (*n* = 85; 64.9%) said that some of their symptoms were made by their minds. Just below a third (*n* = 41; 31.3%) stated that they do not require any medication, and 32.8% (*n* = 43) felt they did not need to be seen by a psychiatrist.

Those participants who had good insight were significantly more likely to report feeling relieved about the admission at discharge (*p* = 0.001). Similarly, those participants who had good insight were more likely to report feeling pleased about the admission at discharge (*p* = 0.037). Those participants with poor insight were more likely to report feeling angry (*p* = 0.041) when compared to those with good insight. There was no significant association between level of insight and perceived coercion (*p* = 0.25), negative pressures (*p* = 0.10) and procedural justice (*p* = 0.056; Table 4).

**Table 4:** The associations between levels of insight and the MAES.

	Level	Poor insight	Good insight	*p*-Value	Test
*N* (131)		48 (36.6%)	83 (63.4%)		
Coercion	Low	10 (21%)	25 (30%)	0.25	Pearson’s chi-squared
	High	38 (79%)	58 (70%)		
Negative pressure	Low	11 (23%)	10 (12%)	0.10	Pearson’s chi-squared
	High	37 (77%)	73 (88%)		
Procedural injustice	High	17 (35%)	44 (53%)	0.052	Pearson’s chi-squared
	Low	31 (65%)	39 (47%)		
Affective Reactions					
Angry	False	22 (46%)	53 (64%)	0.041	Fisher’s exact
	True	26 (54%)	28 (34%)		
	Do not know	0 (0%)	2 (2%)		
Sad	False	19 (40%)	43 (52%)	0.23	Fisher’s exact
	True	29 (60%)	39 (47%)		
	Do not know	0 (0%)	1 (1%)		
Pleased	False	36 (75%)	45 (54%)	0.037	Fisher’s exact
	True	12 (25%)	36 (43%)		
	Do not know	0 (0%)	2 (2%)		
Relieved	False	28 (58%)	23 (28%)	0.001	Fisher’s exact
	True	19 (40%)	57 (69%)		
	Do not know	1 (2%)	3 (4%)		
Confused	False	21 (44%)	52 (63%)	0.064	Fisher’s exact
	True	26 (54%)	30 (36%)		
	Do not know	1 (2%)	1 (1%)		
Frightened	False	27 (56%)	55 (66%)	0.33	Fisher’s exact
	True	21 (44%)	27 (33%)		
	Do not know	0 (0%)	1 (1%)		

## Discussion

This study examined the admission experience of involuntary mental health care users at two psychiatric hospitals in KwaZulu Natal using the MAES (short form) and identified several areas of concern. At the time of the involuntary admission, most participants experienced a subjective sense of being coerced, and stated that pressure or threats were exerted on them by others. Every second patient reported that their opinions regarding the admission process did not matter. Patients therefore reported feeling sad, angry, and confused.

However, at discharge the BIS showed that two thirds of the sample recognized the need for the hospitalization, and more than one third admitted that they were pleased they were admitted. There was a significant association between good insight and a feeling of being relieved, and pleased regarding the admission. Unsurprisingly, there was a significant association between poor insight and feelings of anger. This finding is in contrast to a qualitative study from Greece that reported that 80% of participants felt that objective coercive measures, such as seclusion, were unnecessary and traumatizing (40). Patients have also viewed seclusion as a punishment and felt it was used inappropriately (41). However, these studies looked specifically at the patient’s views on seclusion, not the involuntary admission as a whole.

The findings of this study regarding high levels of coercion and perceived coercion (73%) are in keeping within the range of 59 to 89% reported by international studies. The proportion of participants with high MPCS scores is higher than that reported in studies from Norway (59%) (42), Ireland (39.6%) (19), and Pakistan (21%) (13), but lower than that reported by Sheehan et al. (43) (89%) and Katsakou et al. (44) (87%) from England. The high proportion (84%) of patients who reported threats at the time of admission in this study is concerning because this proportion is much higher than that reported in previous studies from Ireland (19.6%) (19), Pakistan (41%) (13), and England where 48.6% experienced no negative pressure at all (43). The proportion of patients who felt that they were unheard in this study (46.6%), is lower than that reported by O’Callaghan et al. (19) (61%).

Likely explanations for the high proportion of patients who experienced coercion and threats in our study can be explained by the inclusion of voluntary patients in some studies, diagnostic heterogeneity, different cut-off values and the study setting. Perceived coercion on admission has been significantly associated with involuntary admission in many studies (2, 4, 19). All (100%) of the participants in our study were involuntary compared to 39% in the study from Pakistan (13), and 22% in the study from Ireland (19). Most patients in this study had a diagnosis of a psychotic disorder. In the studies that reported lower levels of coercion more patients were diagnosed with mood disorders than with psychotic disorders (19) More than two thirds of the participants in this study had to be sedated on admission because they were aggressive or uncooperative and therefore needed to be coerced into admission. It is therefore possible that the rationale for the involuntary admission of the majority of the participants in this study was aggression. A US survey found that over 60% of respondents perceived people who met the criteria for schizophrenia as a danger to others, and 44–59% supported coercive treatment for such individuals (45). In SA, there is a shortage of ambulance services, with a third of patients being transported to the hospital by the police. It is likely that police services would be more likely to use force to transport patients to the hospital compared to health workers.

The sadness, anger and confusion experienced by the participants in this study confirms that globally an involuntary admission evokes negative emotions (6–12). In contrast with other international studies, this study did not find a significant association between demographic variables such as race, gender, and employment status and the MAES scores. This may be attributed to the smaller sample size in this study. Our findings show that participants with good insight at discharge viewed their involuntary admission experience positively. This is in keeping with the two main narratives identified in previous studies. Involuntary admission was either justified or considered to be a complex emotional experience that infringed on patients’ autonomy and freedom of movement (2).

Coercive interventions in psychiatry are facing increasing criticism because they are emotional, traumatic events for all involved (46). There is global consensus that coercive measures in psychiatry must only be used when there are no other alternatives (47). The strengths of this study are the use of a standardized instrument to describe the patient experience, the inclusion of involuntary patients only and that this study provides information that is lacking in this setting.

This study has several potential limitations. The BIS and MAES (short form) have not been validated for use in our patient population, therefore generalizations of the findings of this study should be applied with caution. Furthermore, other factors that were not investigated in this study may also be responsible for the high levels of coercion experienced by the participants in this study. A selection bias is also possible, because patients who were angry and dissatisfied regarding the involuntary admission were more likely to have not been referred to the principal investigator. Recall bias must also be taken into consideration because participants were interviewed at discharge, and not at admission. Lastly, due to the small sample size, the findings do not represent the entire population from which it was drawn.

This study confirms the findings of previous studies. Most patients were coerced and threatened when they were admitted involuntarily, with little regard for their participation in the process. Negative admission experiences lead to adverse patient outcomes (2). Our results highlight the need for shared decision making to improve the doctor patient relationship. Although acutely psychotic patients may not have the capacity to make informed decisions regarding their admission, it is still possible to communicate and explain the rationale for the admission to them. Also, the capacity for informed consent must be re-evaluated continuously. Wherever possible, once a patient regains capacity, clinicians may consider offering their patients the option of an advanced directive, so that they may decide together what can be done in the event that the patient becomes so severely ill that he/she lacks the capacity to make an informed decision. Further research must be undertaken to identify strategies that are feasible to improve the overall involuntary admission experience in our setting.

## Data availability statement

The raw data supporting the conclusions of this article will be made available by the authors, without undue reservation.

## Ethics statement

The studies involving human participants were reviewed and approved by University of KwaZulu-Natal Biomedical Research Ethics Committee. The patients/participants provided their written informed consent to participate in this study.

## Author contributions

All authors listed have made a substantial, direct, and intellectual contribution to the work and approved it for publication.

## Conflict of interest

The authors declare that the research was conducted in the absence of any commercial or financial relationships that could be construed as a potential conflict of interest.

## Publisher’s note

All claims expressed in this article are solely those of the authors and do not necessarily represent those of their affiliated organizations, or those of the publisher, the editors and the reviewers. Any product that may be evaluated in this article, or claim that may be made by its manufacturer, is not guaranteed or endorsed by the publisher.

## Abbreviations

MAES: The MacArthur Admission Experience Survey
MPCS: The MacArthur Perceived Coercion Scale
MNPS: The MacArthur Negative Pressures Scale
MPJS: The MacArthur Procedural Justice Scale
BIS: Birchwood insight scale
SA: South Africa
MHCA: Mental Health Care Act

## References

[1] Chieze M Clavien C Kaiser S Hurst S. Coercive measures in psychiatry: a review of ethical arguments. Front Psych. (2021) 12:790886. doi: 10.3389/fpsyt.2021.790886, PMID: 34970171 PMC8712490

[2] Iudici A Girolimetto R Bacioccola E Faccio E Turchi G. Implications of involuntary psychiatric admission: health, social, and clinical effects on patients. J Nerv Ment Dis. (2022) 210:290–311. doi: 10.1097/NMD.0000000000001448, PMID: 35349506

[3] Molodynski A Rugkåsa J Burns T. Coercion and compulsion in community mental health care. Br Med Bull. (2010) 95:105–19. doi: 10.1093/bmb/ldq015, PMID: 20501486

[4] Sampogna G Luciano M Del Vecchio V Pocai B Palummo C Fico G. Perceived coercion among patients admitted in psychiatric wards: Italian results of the EUNOMIA study. Front Psych. (2019) 10:316. doi: 10.3389/fpsyt.2019.00316, PMID: 31164841 PMC6536685

[5] Szmukler G Appelbaum PS. Treatment pressures, leverage, coercion, and compulsion in mental health care. J Ment Health. (2008) 17:233–44. doi: 10.1080/09638230802052203

[6] Armgart C Schaub M Hoffmann K Illes F Emons B Jendreyschak J. Negative emotions and understanding - patients' perspective on coercion. Psychiatr Prax. (2013) 40:278–84. doi: 10.1055/s-0033-1343159, PMID: 23633147

[7] Katsakou C Priebe S. Patient's experiences of involuntary hospital admission and treatment: a review of qualitative studies. Epidemiol Psichiatr Soc. (2007) 16:172–8. doi: 10.1017/S1121189X00004802, PMID: 17619549

[8] Wykes T Csipke E Williams P Koeser L Nash S Rose D. Improving patient experiences of mental health inpatient care: a randomized controlled trial. Psychol Med. (2018) 48:488–97. doi: 10.1017/S003329171700188X, PMID: 28726599 PMC5757411

[9] Wyder M Bland R Blythe A Matarasso B Crompton D. Therapeutic relationships and involuntary treatment orders: service users' interactions with health-care professionals on the ward. Int J Ment Health Nurs. (2015) 24:181–9. doi: 10.1111/inm.12121, PMID: 25628260

[10] Murphy R McGuinness D Bainbridge E Brosnan L Felzmann H Keys M. Service Users' experiences of involuntary hospital admission under the mental health act 2001 in the Republic of Ireland. Psych Serv. (2017) 68:1127–35. doi: 10.1176/appi.ps.201700008, PMID: 28669292

[11] Sibitz I Scheutz A Lakeman R Schrank B Schaffer M Amering M. Impact of coercive measures on life stories: qualitative study. Br J Psychiatry. (2011) 199:239–44. doi: 10.1192/bjp.bp.110.087841, PMID: 21778173

[12] Akther SF Molyneaux E Stuart R Johnson S Simpson A Oram S. Patients' experiences of assessment and detention under mental health legislation: systematic review and qualitative meta-synthesis. B J Psych open. (2019) 5:e37. doi: 10.1192/bjo.2019.19, PMID: 31530313 PMC6520528

[13] Zuberi SI Sajid A Yousafzai AW Bhutto N Khan MM. Perceived coercion and need for hospital admission among psychiatric in-patients: figures from a Pakistani tertiary care hospital. Int Psych. (2011) 8:14–6. doi: 10.1192/S1749367600006184, PMID: 31508067 PMC6734997

[14] Hoge SK Lidz C Mulvey E Roth L Bennett N Siminoff L. Patient, family, and staff perceptions of coercion in mental hospital admission: an exploratory study. Behav Sci Law. (1993) 11:281–93. doi: 10.1002/bsl.2370110306, PMID: 10150231

[15] O'Donoghue B Lyne J Hill M O'Rourke L Daly S Larkin C. Perceptions of involuntary admission and risk of subsequent readmission at one-year follow-up: The influence of insight and recovery style Journal of Mental Health (2011). 20, 249–259. 21574790 10.3109/09638237.2011.562263

[16] Klag S Creed P O'Callaghan F. Development and initial validation of an instrument to measure perceived coercion to enter treatment for substance abuse. Psychol Addict Behav. (2006) 20:463–70. doi: 10.1037/0893-164X.20.4.463, PMID: 17176181

[17] Fu JC-K Chow PP-L Lam LC-W. The experience of admission to psychiatric hospital among Chinese adult patients in Hong Kong. BMC Psychiatry. (2008) 8:86. doi: 10.1186/1471-244X-8-86, PMID: 18928557 PMC2596108

[18] Simpson AIF Boldt I Penney S Jones R Kidd S Nakhost A. Perceptions of procedural justice and coercion among forensic psychiatric patients: a study protocol for a prospective, mixed-methods investigation. BMC Psychiatry. (2020) 20:230. doi: 10.1186/s12888-020-02629-6, PMID: 32404082 PMC7222335

[19] O'Callaghan AK Plunkett R Kelly BD. The association between perceived coercion on admission and formal coercive practices in an inpatient psychiatric setting. Int J Law Psychiatry. (2021) 75:101680. doi: 10.1016/j.ijlp.2021.101680, PMID: 33609996

[20] Mielau J Altunbay J Gallinat J Heinz A Bermpohl F Lehmann A. Subjective experience of coercion in psychiatric care: a study comparing the attitudes of patients and healthy volunteers towards coercive methods and their justification. Eur Arch Psychiatry Clin Neurosci. (2016) 266:337–47. doi: 10.1007/s00406-015-0598-9, PMID: 25900468

[21] Raveesh BN Pathare S Lepping P Noorthoorn EO Gowda GS Bunders-Aelen JG. Perceived coercion in persons with mental disorder in India: a cross-sectional study. Indian J Psychiatry. (2016) 58:S210–20. doi: 10.4103/0019-5545.196846, PMID: 28216772 PMC5282618

[22] Xu Z Müller M Lay B Oexle N Drack T Bleiker M. Involuntary hospitalization, stigma stress and suicidality: a longitudinal study. Soc Psychiatry Psychiatr Epidemiol. (2018) 53:309–12. doi: 10.1007/s00127-018-1489-y, PMID: 29380026

[23] Szabo CP Kaliski SZ. Mental health and the law: a south African perspective. B J Psych Int. (2017) 14:69–71. doi: 10.1192/S2056474000001951, PMID: 29093950 PMC5618904

[24] The Presidency Republic of South Africa. The mental health care act no 17 of 2002. Pretoria: The Government Gazette (2002).

[25] Burns JK. Mental health services funding and development in KwaZulu-Natal: A tale of inequity and neglect South African Medical Journal (2010). 100:662–666. doi: 10.7196/SAMJ.4100 21080996

[26] Bartlett P Jenkins R Kiima D. Mental health law in the community: thinking about Africa. Int J Ment Heal Syst. (2011) 5:21. doi: 10.1186/1752-4458-5-21, PMID: 21914182 PMC3189124

[27] Ramlall S Chipps J Mars M. Impact of the south African mental health care act no. 17 of 2002 on regional and district hospitals designated for mental health care in KwaZulu-Natal. South African medical journal =. Suid-Afrikaanse Tydskrif Vir Geneeskunde. (2010) 100:667–70. 21080997 10.7196/samj.4129

[28] Swanepoel M Mahomed S. Involuntary admission and treatment of mentally ill patients – the role and accountability of mental health review boards. South African J Bioethics Law. (2021) 14:84–8. doi: 10.7196/SAJBL.2021.v14i3.717

[29] Madala-Witbooi NJ Adeniyi OV. Demographic and clinical profiles of admitted psychiatric patients of the East London mental health unit in the eastern cape, South Africa. Medicine. (2019) 98:e18399-e. doi: 10.1097/MD.0000000000018399 31876712 PMC6946551

[30] Raphalalani S Becker PJ Böhmer MW Krüger C. The role of mental health care act status in dignity-related complaints by psychiatric inpatients: a cross-sectional analytical study. S Afr J Psychiatry. (2021) 27:1602. doi: 10.4102/sajpsychiatry.v27i0.1602 PMC8182446 34192081

[31] Golay P Morandi S Silva B Devas C Bonsack C. Feeling coerced during psychiatric hospitalization: impact of perceived status of admission and perceived usefulness of hospitalization. Int J Law Psychiatry. (2019) 67:101512. doi: 10.1016/j.ijlp.2019.101512, PMID: 31785727

[32] Kallert TW Glöckner M Onchev G Raboch J Karastergiou A Solomon Z. The EUNOMIA project on coercion in psychiatry: study design and preliminary data. WPA. (2005) 4:168–72. PMC1414770 16633543

[33] Golay P Semlali I Beuchat H Pomini V Silva B Loutrel L. Perceived coercion in psychiatric hospital admission: validation of the French-language version of the MacArthur admission experience survey. BMC Psychiatry. (2017) 17:1519. doi: 10.1186/s12888-017-1519-4 PMC5674868 29110643

[34] Gowda GS Noorthoorn EO Kumar CN Nanjegowda RB Math SB. Clinical correlates and predictors of perceived coercion among psychiatric inpatients: a prospective pilot study. Asian J Psychiatr. (2016) 22:34–40. doi: 10.1016/j.ajp.2016.04.004, PMID: 27520892

[35] Birchwood M Smith J Drury V Healy J Macmillan F Slade MA. A self-report insight scale for psychosis: Reliability, validity and sensitivity to change Acta Psychiatrica Scandinavica (1994). 89, 62–67. doi: 10.1111/j.1600-0447.1994.tb01487.x 7908156

[36] Phahladira L Asmal L Kilian S Chiliza B Scheffler F Luckhoff HK. Changes in insight over the first 24 months of treatment in schizophrenia spectrum disorders. Schizophr Res. (2019) 206:394–9. doi: 10.1016/j.schres.2018.10.013, PMID: 30385130

[37] Asmal L du Plessis S Vink M Fouche J-P Chiliza B Emsley R. Insight and white matter fractional anisotropy in first-episode schizophrenia. Schizophr Res. (2017) 183:88–94. doi: 10.1016/j.schres.2016.11.005, PMID: 27887780

[38] Patridge EF Bardyn TP. Research electronic data capture (REDCap). J Med Libr Assoc. (2018) 106:142–4. doi: 10.5195/jmla.2018.319

[39] Dictionary OE. (2022). Art, n.1 Oxford University Press.

[40] Bilanakis N Peritogiannis V. Attitudes of patients and families toward restraint and seclusion. Psych Serv. (2008) 59:1220. doi: 10.1176/ps.2008.59.10.1220 18832516

[41] Martinez RJ Grimm M Adamson M. From the other side of the door: patient views of seclusion. J Psychosoc Nurs Ment Health Serv. (1999) 37:13–22. doi: 10.3928/0279-3695-19990301-14, PMID: 10098107

[42] Ivar Iversen K Høyer G Sexton H Grønli OK. Perceived coercion among patients admitted to acute wards in Norway. Nord J Psychiatry. (2002) 56:433–9. doi: 10.1080/08039480260389352, PMID: 12495538

[43] Sheehan KA Burns T. Perceived coercion and the therapeutic relationship: a neglected association? Psych Serv. (2011) 62:471–6. doi: 10.1176/ps.62.5.pss6205_0471, PMID: 21532071

[44] Katsakou C Bowers L Amos T Morriss R Rose D Wykes T. Coercion and treatment satisfaction among involuntary patients. Psychiatr Serv. (2010) 61:286–92. doi: 10.1176/ps.2010.61.3.286 20194406

[45] Pescosolido BA BM, and John Monahan. Evolving public views on the likelihood of violence from people with mental illness: stigma and its consequences. Health Aff. (2019) 38:1735–43. doi: 10.1377/hlthaff.2019.00702, PMID: 31589533

[46] Krieger E Moritz S Lincoln TM Fischer R Nagel M. Coercion in psychiatry: a cross-sectional study on staff views and emotions. J Psychiatr Ment Health Nurs. (2021) 28:149–62. doi: 10.1111/jpm.12643, PMID: 32348607

[47] United Nations. (2006). Convention on the rights of persons with disabilities. Treaty Series, 2515:3. 10.1515/9783110208856.203 18348362

